# Inflammation in myocardial infarction: roles of mesenchymal stem cells and their secretome

**DOI:** 10.1038/s41420-022-01235-7

**Published:** 2022-11-09

**Authors:** Lianbo Shao, Ying Shen, Chuanlu Ren, Shuzo Kobayashi, Takayuki Asahara, Junjie Yang

**Affiliations:** 1 Department of Cardiovascular Surgery, The First Affiliated Hospital of Soochow University, Suzhou, 215006 P. R. China; 2 National Clinical Research Center for Hematologic Disease, Jiangsu Institute of Hematology, The First Affiliated Hospital of Soochow University, Suzhou, 215006 P. R. China; 3 The 904th Hospital of CPLA, Wuxi, 214000 P. R. China; 4 Division of Regenerative Medicine, Department of Center for Clinical and Translational Science, Shonan Kamakura General Hospital, Kamakura, Japan

**Keywords:** Mesenchymal stem cells, Regeneration

## Abstract

Inflammation plays crucial roles in the regulation of pathophysiological processes involved in injury, repair and remodeling of the infarcted heart; hence, it has become a promising target to improve the prognosis of myocardial infarction (MI). Mesenchymal stem cells (MSCs) serve as an effective and innovative treatment option for cardiac repair owing to their paracrine effects and immunomodulatory functions. In fact, transplanted MSCs have been shown to accumulate at injury sites of heart, exerting multiple effects including immunomodulation, regulating macrophages polarization, modulating the activation of T cells, NK cells and dendritic cells and alleviating pyroptosis of non-immune cells. Many studies also proved that preconditioning of MSCs can enhance their inflammation-regulatory effects. In this review, we provide an overview on the current understanding of the mechanisms on MSCs and their secretome regulating inflammation and immune cells after myocardial infarction and shed light on the applications of MSCs in the treatment of cardiac infarction.

## Abstract (web summary)

MSCs and their secretome-induced inflammatory modulation.

## Graphical abstract

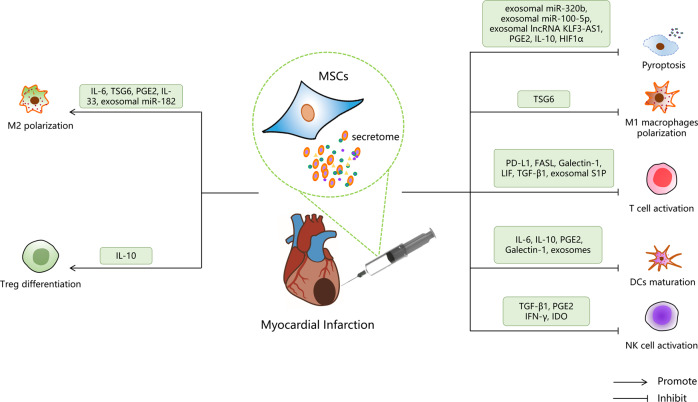

## Facts

- Inflammation is initiated after myocardial infarction (MI) and its duration and intensity determine the prognosis of MI.
- Mesenchymal stem cells (MSCs) transplantation serves as an effective way for MI therapy owing to their immunomodulatory property.
- The paracrine effects rather than differentiation of MSCs are considered to be the main mechanism for enhancing cardiac function.

## Open questions

- What are the major mechanisms of MSCs and their secretome mediated inflammation regulation in MI microenvironment?
- How to improve the immunomodulatory properties of MSCs and boost their cardiac repair functions?

## Introduction

Myocardial infarction (MI) remains a leading cause of death and emerges as a worldwide public health challenge in recent years [1]. At the onset of MI, shortage of blood and oxygen supply undeniably induces the death of cardiomyocytes and other types of cells in the heart. Meanwhile, inflammation is initiated with the inflammatory cell infiltration, accompanied with the activation of both innate and adaptive immune responses [2, 3]. Accumulating reports have demonstrated the dual effects of inflammation on the cardiac repair [4], that is, clearing dead cells and debris in one way, and exacerbating damage if the inflammation is prolonged in the other way. In fact, cardiac inflammation is manipulated exquisitely by diverse crucial inflammatory cytokines and potentially reversible by regulating some of these factors [5], making it as a promising target for enhancing cardiac function.

Mesenchymal stem cells (MSCs) have attracted much attention for their therapeutic potentials such as anti-apoptosis, immunomodulation, anti-inflammation, pro-angiogenesis and regeneration. Undoubtedly, MSCs transplantation is a safe and promising approach for MI therapy. Originally, direct differentiation to functional cells, such as endothelial cells, was considered to be the main mechanism for MSCs-mediated cardiac therapy [6]. However, accumulating evidence indicated that MSCs conduct repair function through alternative modes including paracrine secretion of growth factors and inflammation-regulatory factors rather than engraftment and differentiation [7, 8]. Here, we summarized current knowledge regarding the mechanisms of MSCs and their secretome mediated inflammation regulation for rejuvenating the infarcted myocardium. We also outlined various approaches to enhance the curative effects of MSC-based therapy for MI patients.

## Inflammatory response determines the prognosis of MI

Massive cell death occurs at the onset of infarction, which induces a drastic inflammation that is essential for cardiac repair, but is also involved in the pathogenic process of ventricular remodeling. Inflammation is initiated to clear necrotic cell debris and extracellular matrix (inflammatory phase), then followed with fibroblasts transdifferentiation and scar formation (reparatory phase). However, the duration and intensity of the inflammatory response determine the prognosis of MI. Extensive experimental investigations demonstrated that prolonged and excessive inflammation resulted in accentuated injury and increased left ventricular adverse remodeling following MI [9–12]. Therefore, maintaining an appropriate and timely homeostasis balance between inflammatory and reparatory phases is crucial for the regeneration of the injured myocardium [13].

Over the last three decades, relentless efforts targeting at either reduction of the inflammatory pathways or activation of reparatory pathways have been performed in the aim of improving the prognosis of MI [14, 15]. Attenuation of inflammation can be achieved through inhibition of leukocytes activation, modification of macrophages polarization toward reparative phenotype, or deactivation of regulatory T cells [12, 16]. Unfortunately, most of them didn’t achieve satisfactory results. As an example, clinical trials by Sheehan group and Yu group showed that targeting integrins to reduce leukocyte activation in MI patients did not demonstrate statistically significant differences in infarcted size compared to control patients [17, 18]. Till now, there has been no effective immunoregulatory or inflammation-regulatory therapeutic strategies used in large-scale clinical practice for MI treatment [19–21]. As we know, the cell types are various and the microenvironment is spatially heterogeneous in the remodeling heart, targeting a specific inflammatory factor or signal pathway is thus not able to achieve great effectiveness [10, 22]. Therefore, fine tuning of the inflammatory microenvironment is key to the success of myocardial repair.

## MSCs and their secretome for immune and inflammation regulation

It has been demonstrated stem cell-based therapy is a promising alternative strategy for MI therapy [23]. Several types of stem cells including embryonic stem cells (ESCs), mesenchymal stromal/stem cells (MSCs), induced pluripotent stem cells (iPSCs) and endothelial progenitor cells (EPCs) have been verified to be effective for cardiac repair. Among these cell types, MSCs gained more attention in tissue regeneration because of their self-renewal, extensive differentiation ability and immunomodulatory properties [24, 25]. In animal models or clinical trials of MI, MSCs transplantation has been demonstrated to confer benefits on the restoration of heart functions. According to recent investigations, the immunomodulatory property rather than the differentiation ability of MSCs accounts for their therapeutical effects [26]. Specifically, MSCs exert their inflammation-regulating roles mainly relying on: (i) direct cell to cell communication; (ii) paracrine of the immunomodulatory secretome [27]. They could interact with almost all of the immune cells and modulate their functions, for example, regulating the function of lymphocyte subpopulations (CD8+ T cells, regulatory T cells, NK cells), modulating macrophages polarization from pro-inflammatory M1 phenotype toward regenerative M2 phenotype, and adjusting the maturation and function of dendritic cells (DCs) [28–31].

The paracrine effects are considered to be the main mechanism for their immune regulation [32, 33]. Presenting into an inflammatory environment, MSCs can orchestrate local inflammatory responses through the release of secretome, which contains various growth factors, inflammatory regulators and extracellular vesicles (such as exosomes) [25, 34]. For example, MSCs secreted a high level of anti-inflammatory cytokine Interleukin (IL)-6 to regulate macrophage polarization [35]. In addition, prostaglandin E2 (PGE2) secreted from MSCs plays multiple roles in alleviating inflammation, such as inhibiting the polarization of M1 macrophages and the maturation and antigen-presenting function of DCs, and alleviating the inflammatory functions of T cells. To be noted, decreasing the expression of PGE2 in MSCs abolished their therapeutic effects [35–37]. Moreover, leukemia inhibitory factor (LIF) and hepatocyte growth factor (HGF), secreted by MSCs, have been shown to inhibit the differentiation of Th1 and Th17 cells [38]. To be specific, LIF plays a vital role in inducing Th17 cells differentiation through increasing the expression of ERK and downregulating the activation of STAT3, whereas HGF inhibits the proliferation of activated peripheral blood mononuclear cells (PBMCs) by enhancing cell apoptosis [39]. Interestingly, stimulation of MSCs with pro-inflammatory factors such as TNF-α or IL-1β can change the secretome and augment the regenerative capacity of the cells [40]. In fact, their immune regulatory function can be enhanced when exposed to an inflammatory microenvironment, namely ‘MSCs licensing’ [41]. For example, TNFα-stimulated MSCs could release more TNFα-stimulated gene-6 (TSG6, a secreted 30 kD anti-inflammatory factor), and induce macrophages polarized from M1 phenotype toward M2 phenotype via suppressing the activation of NF-κB pathway [42].

A decade ago, Lai et al. transplanted the conditioned media of MSCs into infarcted myocardium and proved the cardioprotective effects [33]. Further studies have demonstrated that exosomes in MSC secretome exerted the cardioprotective effects, in contrast, the depletion of exosomes from the conditioned media abolished the effects. Recently, exosomes are more and more recognized as important intercellular mediators by transferring biological signals, instead of the original thought to be wasted materials disposed by cells. Some groups have demonstrated that MSC-derived exosomes (MSC-Exo), transplanted by intramyocardial injection, can reduce infarct size and preserve heart function via anti-apoptosis, pro-angiogenesis and immune regulation, acting similarly as MSCs [43]. Moreover, intravenously-administered MSC-Exo decreased the filtration of neutrophils in the infarcted heart, as well as decreased the quantity of M1 macrophages in the acute phase after MI [44, 45].

Overall, these results (Table 1) suggest that paracrine effects of MSCs are essential and crucial in the regulation of inflammation, listing the cell as an ideal candidate for cardiac tissue regeneration post MI.

**Table 1:** Paracrine effectors of MSCs in the regulation of inflammation.

Secretome	The mechanisms for regulating inflammation	Reference
IL-6	Promote IL-6-dependent M2 polarization	[35]
PGE2	Inhibit M1 macrophages polarization, DCs maturation, and alleviate the inflammatory functions of T cells	[35–37]
LIF	Inhibit the differentiation of Th1 and Th17 cells	[38, 39]
HGF	Inhibit expansion of activated PBMCs by promoting their apoptosis	[38, 41]
Exosomes	Decrease the filtration of neutrophils and reduce the ratio of M1 macrophages	[44, 45]

## MSCs and their secretome regulate macrophage polarization

Under steady physiological conditions, cardiac resident macrophages maintain homeostasis of the heart by removing senescent or damaged cells [46]. At the onset of myocardial infarction, sterile inflammation is initiated by pro-inflammatory cytokines increasingly released into the microenvironment. In response to this injury, circulating monocytes infiltrate in the ischemic myocardium and differentiate into M1 macrophage, scavenging the necrotic cells debris and releasing pro-inflammatory cytokines. M1 macrophages can then transform into anti-inflammatory M2 phenotype, thereby alleviating the inflammatory response and initiating cardiac repair [47, 48]. M1 macrophage and M2 macrophage can be dynamically interchanged in response to the alterations of the inflammatory microenvironment. Thus, regulating the polarization of macrophages can be used to alleviate inflammation and promote repair of damaged tissue.

Accumulating evidence has indicated that MSCs contribute to cardiac repair through modulating the functions of macrophages [45, 49, 50]. Ben-Mordechai et al. demonstrated the MSC-mediated cardiac repair was diminished with macrophage depletion, resulting in increased infarct size and a significant incidence of cardiac remodeling [51]. In addition, injection of MSCs into the ischemic myocardium can induce the enhanced expression of Arg1 and CD206 (M2 markers) in macrophages without altering the total cell number, suggesting the phenotype shift from M1 to M2 [52, 53]. Recently, increasing evidence has demonstrated that MSCs can transform the polarization and migration of macrophages through their secreted factors and exosomes [45, 54]. Zhao et al. demonstrated that TSG6, secreted by MSCs, can induce polarization of pro-inflammatory M1 phenotype toward regenerative M2 phenotype via suppressing NF-κB pathway, and promotes regeneration of injured tissues in a mouse model [42]. Notably, TSG6 can also inhibit the migration of macrophages to injured and inflamed sites via binding to CD44 [55]. In addition, MSCs, activated by damage associated molecular patterns (DAMPs), can secrete PGE2, which converts macrophages from M1 macrophage polarized into regenerative phenotype [56]. A recent study by Chen et al. overexpressed IL-33 in MSCs and showed that IL-33 overexpression enhanced the immunomodulatory function and therapeutic effects of the cells via facilitating M2 macrophages polarization [57]. The recent findings on MSC-derived exosomes also highlighted potential mechanisms on the regulation of macrophage polarization [45, 58, 59]. For instance, miR-182, contained in MSC-Exo, can preserve heart function via inhibiting the expression of TLR4 and enhancing M2 polarization [45]. Taken together, the polarization of macrophages is essential for cardiac healing and repair, which can be modulated by MSCs via their secretome (Table 2).

**Table 2:** Paracrine effectors of MSCs in the regulation of macrophage polarization.

Factors	Effectiveness	Mechanisms	Reference
TSG6	Induce M2 macrophages polarization	Suppress NF-κB pathway activation	[42]
TSG6	Inhibit macrophages migration	Bind to CD44	[55]
PGE2	Convert macrophages into regenerative phenotype	Not mentioned	[56]
IL-33	Enhance M2 macrophages polarization	Not mentioned	[57]
miR-182	Convert M1 macrophages to M2 phenotype	Inhibit TLR4 expression	[45]

## MSCs regulate activation of T cells and NK cells after MI

Inflammation is a complex biological response that involves innate and adaptive immune responses in the context of MI [3, 60–62]. T cells, as effector cells in adaptive immune responses, participate in the pathological process after MI [3, 61]. Based on the different functions and surface markers, T cells are divided into cytotoxic T cells (CD8+), T helper cells (CD4+), Treg cells (CD4+CD25+ FoxP3+), NK T cells and so on [63, 64]. Both cytotoxic T cells and T helper cells recognize specific antigens, and then cytotoxic T cells induce the apoptosis of the cells that present the specific antigens directly. Treg cells play immunoregulatory roles and terminate T cell-mediated immune response. Specifically, Treg cells suppress proliferation and activation of target T cells through secreted inhibitory cytokines including IL-10 and TGF-β. NK cells also participate in the modulation of innate and adaptive immune responses by secreting cytokines such as IFN-γ. In the microenvironment of MI, NK cells can recognize and lyse the stressed cells stimulated with ischemia [65]. Considering the functions of T cells, regulation of different T cells populations contributes to the modulation of inflammation process in the ischemic myocardium.

MSCs can modulate T cells by the direct contact to T cells and paracrine effects. PD-L1 and FASL are the two main molecules on the membrane of MSCs that are involved in regulation of T cells. As a mechanism of action, programmed cell death protein (PD)-L1 on MSCs directly binds to PD-1 or CD80 expressed on T cells, and delivers an inhibitory signal to inhibit their proliferation [66]. Furthermore, pretreatment of MSCs with IFN-γ can increase their ability of inhibiting T proliferation through upregulation of PD-L1. Similarly, Fas ligand (FASL) on MSCs membrane induces apoptosis of T cells by interacting with FAS on T cells [67]. Galectin-1, a member of the galactose agglutination family, is also abundantly expressed on the surface of MSCs and regulates inflammation. Knockdown of their expression in MSCs can enhance the cytotoxicity of T cells and restore the proliferation of CD4+ and CD8+ T cells [68]. Except for these critical cell-surface molecules, MSCs can also regulate T cells through paracrine effects. It has demonstrated that by releasing TGF-β1, human umbilical cord MSCs can inhibit expansion and activation of T cells via arresting them in G_0_ phase through TGF-β signal pathway [69, 70].

Recently, Treg cells have been verified to have the ability for tissue repair and regeneration due to that they can alleviate or shorten the pro-inflammatory phase and initiate the anti-inflammatory phase at the injured tissues. Paracrine factors released by Treg cells can elicit the alteration of macrophages from inflammatory to reparative phenotype, which results in a better myocardium healing and preserved heart function [71, 72]. Accumulating studies showed that MSCs secretome participates in the modulation of Tregs [38, 39, 73]. Li et al. reported that sphingosine 1-phosphate (S1P) in MSC-Exo modulates the expression of Foxp3 in CD4+ T cells, further determines the balance of immunosuppressive Tregs and inflammatory Th17 cells [73]. In addition, the presence of IL-10 in MSCs secretome promoted the expansion of Treg cells by inducing kynurenine (KYN) expression. In line with this, MSC-Exo also prevented conversion of Tregs in Th1/Th17 cells and significantly increased Tregs/Th17 cell ratio depending on indoleamine 2,3-dioxygenase (IDO-1)/KYN manner. Moreover, LIF, secreted by MSCs, has been shown to inhibit the differentiation of Th1 and Th17 cells through activation of ERKs signal and deactivation of STAT3 signal [38, 39] (Table 3).

**Table 3:** Paracrine effectors of MSCs in the regulation of T cells and NK cells.

Factors	Effectiveness	Mechanisms	Reference
PD-L1	Inhibit T cells proliferation	Interact with PD-1 on T cells	[66]
FASL	Induce T cells apoptosis	Interact with FAS on T cells	[67]
Galectin-1	Downregulate inflammation	Inhibit cytotoxicity of T cells and restore the proliferation of CD4+ and CD8+ T cells.	[68]
TGF-β1	Inhibit T cells activation	Activate TGF-β signal pathway and arrest T cells in G0 phase	[69, 70]
S1P in MSCs-Exo	Regulate the balance of Tregs and Th17 cells	Regulate expression of Foxp3 in CD4+ T cells	[73]
IL-10	Promote the expansion of Treg cells	Induce KYN expression	[38]
LIF	Induce Th17 cells differentiation	Activate ERKs signal and deactivate STAT3 signal	[39]

MSCs can also block activation of NK cells and exert a profound inhibitory cytolytic function through multiple cytokines such as TGF-β1, PGE2 and IFN-γ. In addition, by secreting IDO and PEG2, MSCs can reduce the activating receptors (such as NKG2D, NKp44 and NKp30) expression in NK cells [74]. Moreover, transplantation of hiPSC-MSCs decreased the number of NK cells, facilitated the survival of intramyocardially transplanted hiPSC-derived cardiomyocytes, and attenuated the progressive deterioration in left ventricle adverse remodeling [75].

## MSCs regulate activation of dendritic cells after MI

Dendritic cells (DCs) have a strong antigen-presenting capacity and play important roles in orchestrating innate inflammatory responses and adaptive immunity. Mature DCs present antigen to immune cells and activate them in inflammatory microenvironment [76]. After AMI, cardiac antigens are released from the necrotic myocardium, DCs present these antigens to T cells. Then, adaptive immune responses were initiated against the heart [77]. Several studies demonstrated that there is an increase of DCs in the infarcted zone of heart in rat AMI models, and attenuation of DC functions improved heart functions after MI [78–80], which indicated the interventions aimed at controlling DCs numbers or functions has therapeutic potential. It has been demonstrated that MSCs can inhibit T cells activation and expansion through repressing the maturation and function of DCs. For instance, pretreatment of DC progenitors with MSCs blocked the cell differentiation into mature DCs, further leading to a reduced expression of CD80 and CD86, which are necessary to the activation of T cells [81, 82]. In addition, paracrine factors derived from MSCs such as IL-6, IL-10 and exosomes also suppress the maturation of bone marrow-derived DCs, and further alleviate DC-induced immune responses [83]. Moreover, PGE2 and galectin-1 secreted from BMSCs are both considered to be responsible for inhibiting DC cells functions [36, 84]. It has been revealed that pretreatment of DCs with MSCs before transplantation results in decreased homing to lymphoid organs, as well as decreased presentation of antigens to T cells in vivo [85]. Taken together, although there is no direct evidence that MSCs can modulate the functions of DCs in the infarcted heart, it can be speculated that MSCs exert their immunoregulatory function probably through regulating DCs after MI.

## MSCs secretome alleviates pyroptosis of non-immune cells post MI

In addition to these immune cells, MSCs and their secretome also act on non-immune cells including cardiomyocytes and endothelial cells which are also involved in the inflammation after MI. Pyroptosis, as a novel type of programmed cell death, is triggered by multiple pathological stimuli such as inflammation and oxidative stress [86]. Several recent studies demonstrated that pyroptosis can be observed in myocardium post MI, and responsible for the loss of cardiomyocytes after MI [87, 88]. As for the mechanism, pyroptosis is mainly initiated by inflammasomes and executed by caspases and the gasdermin protein family, inducing the secretion of inflammatory cytokines including IL-1β and IL-18 and eventually aggravating the cardiac inflammation [89, 90]. As an important inflammatory pleiotropic cytokine, IL‐1β can activate and recruit monocytes, macrophages and neutrophils to injury sites, and modulates the activation of Th1 and Th17 cells [91]. Meanwhile, IL-18 is important for the cytolytic activity of NK cells and may be involved in the activation of T cells toward Th1 or Th2 cells [92]. Thus, inhibition of pyroptosis signaling pathways provides a promising approach to reduce secondary loss of cardiomyocytes.

MSC-derived exosomes have been confirmed to play vital roles in the regulation of pyroptosis after MI. With the administration of MSC-Exo, the accumulation of inflammatory cells around the infarct zone, and the expression of NLRP3 inflammasome, Caspase-1, gasdermin D (GSDMD) were dramatically reduced [93]. Intriguingly, it has been verified that MSC-Exo suppressed pyroptosis of cardiac cells via delivery of specific ncRNAs [94]. For example, MSCs can inhibit pyroptosis by transporting exosomal miR-320b into cardiomyocytes [95]. In addition, MSC-Exo can deliver miR-100-5p into hypoxia/reoxygenation-injured cardiomyocytes and deactivate NLRP3 inflammasome, inhibiting the release of IL-1β and IL-18 [96]. Interestingly, lncRNA Krüppel-like factor 3 antisense RNA 1 (KLF3-AS1) in MSC-Exo acts as a competing endogenous RNA to sponge miR-138-5p and further regulates the expression of Sirt1, which eventually inhibits cardiomyocytes pyroptosis and attenuates the progression of MI [97]. In addition, MSC-Exo can also alleviate ischemia-induced pyroptosis of endothelial cells and tissue injury [98].

Notably, accumulating evidence also suggests that cytokines and growth factors secreted by MSCs are involved in the regulation of pyroptosis after MI. For instance, PGE2 and IL-10 secreted by MSCs can reduce the function of NLRP3 inflammasome and attenuate pyroptosis [49, 99]. What’s more, Hu et al. found HIF-1α secreted by MSCs can inhibit oxygen and glucose deprivation (OGD)-induced pyroptosis of microglial cells [100]. We have summarized the mechanisms of MSC secretome-regulated pyroptosis in ischemic myocardium in Table 4.

**Table 4:** Paracrine effectors of MSCs in regulation of pyroptosis.

Factors	Effectiveness	Mechanisms	Reference
Exosomal miR-320b	Inhibit pyroptosis	Reduce NLRP3 expression	[95]
Exosomal miR-100-5p	Inhibit NLRP3 inflammasome activation	Decrease FOXO3 expression	[96]
Exosomal lncRNA KLF3-AS1	Inhibit apoptosis and pyroptosis	Act as a ceRNA to sponge miR-138-5p and regulate Sirt1 expression	[97]
PGE2 and IL-10	Inhibit pyroptosis	Reduce the function of NLRP3 inflammasome	[49, 99]
HIF-1α	Inhibit OGD induced cell pyroptosis	Not mentioned	[99, 100]

## Preconditioning of MSCs to enhance their immunomodulatory effects

After MSCs are transplanted into ischemic hearts, they face the harsh microenvironment that discourages their survival and regulatory functions of the local immune responses. Taking this into consideration, preconditioning of MSCs is important to prepare the cells prior to their use in therapy. Preconditioning of MSCs by hypoxia, inflammatory stimulus, or cultured in three-dimension (3D) are all proved to be useful strategies to enhance the therapeutic efficacy of MSCs (Table 5).

**Table 5:** Precondition of MSCs to enhance their immunomodulatory function.

Precondition	Factors	Effectiveness	Reference
Hypoxia	HIF-1α	Impair DCs differentiation, and reduce NK cell-mediated cell lysis	[101]
	miR-146a-5p	Enhance the immunoregulatory effects	[102]
Immunomodulatory factors	IFN-γ	Suppress activated T cells and stimulate Treg	[103]
		Upregulate the expression level of PD-L1 in monocytes	[104]
	TGF-β and IL-6	Enhance adhesion of MSCs through IL-6/ SHP2/integrin signaling axis	[105]
3D culture	Exosomes	Exert greater inhibitory influence on PBMC proliferation.	[106]
	PGE2 and TSG6	Suppress LPS-stimulated macrophages polarization	[107, 110]

### Preconditioning with hypoxia

Previous studies showed preconditioning of MSCs with hypoxia increases their paracrine and immune functions. For example, HIF-1α, produced by hypoxic preconditioned MSCs, can impair the differentiation of DCs, and reduce NK cell-mediated cell lysis [101]. The study by Mao et al. showed pretreatment of MSCs with hypoxia enhanced their immunoregulatory effects and attenuated the inflammation of allergic airway by increasing exosomal miR-146a-5p [102].

### Pretreatment with immunomodulatory factors

As we discussed above, MSC licensing with immunomodulatory factors, especially pro-inflammatory factors, could enhance their immunomodulatory function. For example, T cell-derived IFN-γ could enhance the immunoregulatory ability of MSCs, and in turn lead to suppression of T cell stimulation [103]. In addition, microvesicles secreted from IFN-γ primed MSCs could upregulate the mRNA expression level of PD-L1 in monocytes as well as the ratio of PD-L1, thus reducing immune response in a mouse model of skin graft [104]. Moreover, pretreatment of MSCs with TGF-β only or combined with IL-6 showed a stronger cardioprotective effect by enhancing cell adhesion through the IL-6/integrin signaling axis [105]. In summary, pretreatment of MSCs with immunomodulatory factors could boost MSCs to facilitate required effects.

### 3D culture

Three-dimensional (3D) scaffolds possess a significant advantage for stem cell-based therapies, to our interest, enhancing the survival of MSCs and promoting their immunoregulatory effects. The advantage of the 3D culture is that it supplies a niche and mimics tissue-like structures more effectively than the monolayer cultures. Indeed, exosomes derived from 3D-cultured MSCs exerted a greater inhibitory effects on the PBMCs proliferation than those derived from the 2D culture [106]. Moreover, MSCs cultured in 3D format also showed significantly more effectiveness in suppressing inflammation in animal inflammatory diseases models [107–109]. To deepen our understanding why 3D-cultured MSCs are superior to 2D-cultured ones, mechanism studies have been performed. Several studies have shown that MSCs cultured in 3D format are more effective in the suppression of LPS-stimulated macrophages by producing more PGE2 and TSG6 as compared to 2D culture [107, 110]. Further investigations showed the secretome derived from 3D-cultured MSCs has a plenty amount of anti-inflammatory cytokines such as IL-10 and LIF. In addition, Yan et al. reported that MSCs cultured in 3D secreted more exosomes with regenerative potential compare to those cultured in a conventional 2D environment [111].

## Perspectives

MSCs have been broadly used as a new therapeutic paradigm to treat myocardial infarction. However, there were still a few obstacles limiting the clinical therapeutic application of MSCs. For example, the inflammatory microenvironment in the acute phase of MI resulted in low survival and poor engraftment of transplanted MSCs. In addition, the variance in MSCs donors, culture conditions, number of injected cells and injection routes led to different outcomes. In previous clinical trials, several investigations have reported the significant functional variations of MSCs isolated from different donors or tissues such as bone marrow (BM), adipose tissue (AT), and umbilical cord (UC) [112]. Further investigations suggested the functional variations might be due to that different MSCs emphasized a heterogenic response to inflammatory stimuli [113]. With TNFα priming, BM-MSCs expressed highest levels of SDF1 and VCAM-1, in contrast, AT-MSCs exhibited a prominent upregulation of IDO-1 and CCL5, and UC-MSCs released high levels of IL-6, IL-8, MCP-1 [113]. In a mouse emphysema model, BM-MSCs, AD-MSCs and lung tissue-derived MSCs were administered and the results showed both BM- and AD-MSCs reduced the number of M1 macrophages, in addition, BM-MSCs increased M2 macrophages by changing M1 macrophages into M2 macrophages [114]. Further study showed although different MSC types exhibited similar morphology and surface marker expression, the cytokine profiles of those MSCs are discrepant [115]. All of these factors should be considered before their clinical application for MI treatment.

Approaches targeting leukocyte activation/adhesion, macrophages polarization, or T cells activation are successful in experimental studies to attenuate ischemic injury, leading to considerable enthusiasm for MSC-based MI therapy [12, 16]. Recently, increasing evidence has demonstrated that administration of MSC-sourced secretome is an effective way to fine tune inflammation in MI. The beneficial effects of MSC secretome rely on the cytokines, immunomodulatory factors, growth factors or ncRNA that can be delivered to target cells. Furthermore, it will not trigger allogeneic immune responses and inflammation, in addition to the capacity of long-term preservation and immediately availability. Therefore, in the future experimental and clinical studies, MSC secretome will be an interesting target that deserves further investigations.

## Conclusion

Current medical therapeutic treatments toward MI have not been able to completely reverse the heart failure post MI. Therefore, novel therapeutic strategies are intensely urgent for clinical use. Experimental and clinical studies have demonstrated that MSCs based inflammatory regulation therapy is an effective strategy for myocardial infarction. However, the major setback is low survival and retention of the cells after intramyocardial engraftment, limiting their clinical use. Secretome derived from MSCs plays similar inflammation inhibitory function and cardioprotective effects, and possesses several merits which MSCs lacked. Here, we summarize the current understanding about the mechanisms of MSCs and their secretome mediated inflammation-regulatory function for rejuvenating the heart post MI. MSCs secretome based inflammatory regulation for myocardial infarction pivots from MSC-based cell transplantation to cell-free therapeutics.
